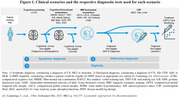# Computerized decision support to optimally funnel patients through the diagnostic pathway

**DOI:** 10.1002/alz.091318

**Published:** 2025-01-09

**Authors:** Aniek M. van Gils, Antti Tolonen, Argonde C. van Harten, Juha Koikkalainen, Frederik Barkhof, Sanna‐Kaisa Herukka, Steen G. Hasselbalch, Patrizia Mecocci, Anne Remes, Hilkka Soininen, Afina Willemina Lemstra, Charlotte Teunissen, Jyrki Lötjönen, Wiesje M. van der Flier, Hanneke F.M. Rhodius‐ Meester

**Affiliations:** ^1^ Alzheimer Center Amsterdam, Neurology, Vrije Universiteit Amsterdam, Amsterdam UMC location VUmc, Amsterdam Netherlands; ^2^ Amsterdam Neuroscience, Neurodegeneration, Amsterdam Netherlands; ^3^ Combinostics Ltd, Tampere Finland; ^4^ University College London, London UK; ^5^ Amsterdam UMC, Amsterdam Netherlands; ^6^ Institute of Clinical Medicine, University of Eastern Finland, Kuopio Finland; ^7^ Danish Dementia Research Centre, Dept. of Neurology, Copenhagen University Hospital ‐ Rigshospitalet, Copenhagen Denmark; ^8^ Division of Clinical Geriatrics, NVS Department, Karolinska Institutet, Stockholm Sweden; ^9^ Institute of Gerontology and Geriatrics, Department of Medicine and Surgery, University of Perugia, Perugia Italy; ^10^ Unit of Clinical Neuroscience, Neurology and Medical Research Center, University of Oulu, Oulo Finland; ^11^ Alzheimer Center Amsterdam, Neurology, Vrije Universiteit Amsterdam, Amsterdam UMC, Amsterdam Netherlands; ^12^ Department of Clinical Chemistry, Neurochemistry Lab and Biobank, Amsterdam Neuroscience, Amsterdam UMC, Location VUmc, Amsterdam Netherlands; ^13^ Department of Epidemiology and Data Science, Vrije Universiteit Amsterdam, Amsterdam UMC, Amsterdam Netherlands; ^14^ Geriatric Medicine, The Memory Clinic, Oslo University Hospital, Oslo, Oslo Norway; ^15^ Internal Medicine, Geriatric Medicine Section, Amsterdam Cardiovascular Sciences Institute, Amsterdam UMC location VUmc, Amsterdam, Noord‐Holland Netherlands

## Abstract

**Background:**

The increasing dementia prevalence and potential introduction of disease‐modifying therapies (DMTs) highlight the need for efficient diagnostic pathways. Clear recommendations to guide the choice of diagnostic tests are lacking and may vary depending on different clinical scenarios. We used a data‐driven approach to identify efficient and effective stepwise diagnostic testing for three clinical scenarios: 1) syndrome diagnosis, 2) etiological diagnosis, 3) potential eligibility for DMT.

**Method:**

We used data from two memory clinic cohorts (ADC, PredictND), including 504 patients with dementia (302 Alzheimer’s disease, 107 frontotemporal dementia, 35 vascular dementia, 60 dementia with Lewy bodies), 191 patients with mild cognitive impairment, and 188 cognitively healthy controls (CN). Tests included digital cognitive screening (cCOG), neuropsychological and functional assessment (NP), MRI with automated quantification, and CSF biomarkers. Sequential testing followed a predetermined order (Figure 1). Subsequent tests were conducted if the diagnosis remained uncertain. Diagnostic certainty was ascertained through a data‐driven clinical decision support system (CDSS) that generated a disease state index probability score (DSI, 0‐1), indicating the probability of each diagnosis. Diagnosis was confirmed if the DSI exceeded a predefined threshold, set based on sensitivity/specificity cutoffs relevant for each clinical scenario and step. We assessed correct diagnoses and the need for additional testing at each step.

**Result:**

For syndrome diagnosis, stepwise testing (cCOG, NP, MRI) accurately identified 71% of the patients, with NP needed in 42%, and MRI in 31%. For etiological diagnosis, starting with cognitive testing to rule out dementia resulted in the need for MRI in 84% of cases, including 91% of dementia patients and 25% CN. Subsequent MRI reduced CSF required to 29%, ultimately diagnosing 81% of patients with 71% accuracy. In determining DMT eligibility, stepwise testing (cCOG, NP, MRI) correctly identified 91% of potential eligible patients for confirmatory CSF testing, while only 51% of ineligible patients.

**Conclusion:**

Depending on the setting, alternative diagnostic pathways are accurate and efficient. As such, a data‐driven tool can assist clinicians in selecting tests of added value across different clinical contexts. This becomes especially important with DMT availability, where the need for more efficient diagnostic pathways is crucial to maintain accessibility and affordability of diagnoses.